# The effects of oxygen tension and antiaging factor *Klotho *on Wnt signaling in nucleus pulposus cells

**DOI:** 10.1186/ar3830

**Published:** 2012-05-02

**Authors:** Akihiko Hiyama, Fumiyuki Arai, Daisuke Sakai, Katsuya Yokoyama, Joji Mochida

**Affiliations:** 1 Department of Orthopaedic Surgery, Surgical Science, Tokai University School of Medicine, 143 Shimokasuya, Isehara, Kanagawa 259-1193, Japan; 2 Research Center for Regenerative Medicine, Tokai University School of Medicine, 143 Shimokasuya, Isehara, Kanagawa 259-1193, Japan

## Abstract

**Introduction:**

The goals of this study were to examine the oxemic regulation of Wnt signaling to explore whether Wnt signaling accelerates the age-related degeneration of nucleus pulposus cells, and if so, to define the mechanism underlying this effect. We investigated the expression of *Klotho*, a newly identified antiaging gene, and whether its regulation is attributable to the suppression of Wnt signaling.

**Methods:**

Rat nucleus pulposus cells were cultured under normoxic (21% O_2_) or hypoxic (2% O_2_) conditions, and the expression and promoter activity of Wnt signaling and *Klotho *were evaluated. The effect of Klotho protein was examined with transfection experiments, 3-(4,5-dimethylthiazol-2-yl)-2,5-diphenyltetrazolium bromide assay, senescence-associated β-galactosidase staining, and cell-cycle analysis. To determine the methylation status of the Klotho promoter region, bisulfite genomic sequencing analysis was performed. Its relation with the activation of Wnt signaling was assessed. We also examined whether the expression of Klotho could block the effects of pathological Wnt expression in nucleus pulposus cells.

**Results:**

Nucleus pulposus cells exhibited increased β-catenin mRNA and protein under the hypoxic condition. Klotho protein was expressed *in vivo*, and protein and messenger RNA expression decreased under the hypoxic condition. Klotho treatment decreased cell proliferation and induced the quiescence of nucleus pulposus cells. In addition, Klotho treatment inhibited expression of β-catenin gene and protein compared with untreated control cells.

**Conclusions:**

These data indicate that Wnt signaling and Klotho form a negative-feedback loop in nucleus pulposus cells. These results suggest that the expression of Klotho is regulated by the balance between upregulation and downregulation of Wnt signaling.

## Introduction

Regenerative therapy for intervertebral disc degeneration has been recently reported [1-3]. Cell-based therapies for tissue regeneration offer an attractive alternative to current conservative, surgical, pharmaceutical, or gene-therapy interventions. However, to clarify the mechanism underlying low-back pain, the molecular mechanisms involved in intervertebral disc degeneration must be identified.

Wnt/β-catenin (hereafter called Wnt) signaling is thought to be involved in the maintenance and destruction of bone and cartilage. Dysregulation of members of this signaling family has been described in osteoarthritis [4-6]. Wnts are secreted glycoproteins crucial for the development and homeostatic renewal of many tissues and for chondrocyte and osteoblast development. In the presence of Wnt ligands, Wnts activate a number of different signaling pathways via distinct receptors and downstream effectors that mediate effects on gene transcription [7-9]. Although Wnt signals regulate the balance between catabolic factors and anabolic factors in intervertebral discs [10,11], their regulation (upstream or downstream) in nucleus pulposus cells and the corresponding signaling mechanisms are unknown.

One of the primary causes of intervertebral disc degeneration is thought to be failure of the nutrient supply to intervertebral disc cells because of structural changes to the cartilage endplate [12]. The O_2 _levels in the nucleus pulposus may be 1% to 5%, and disc-cell metabolism can vary with O_2 _concentration. The activity of disc cells is very sensitive to changes in extracellular oxygen and pH. However, a little information is known about the effect of oxygen tension on nucleus pulposus cells [13]. Accordingly, more data are needed to determine whether a low oxygen tension is beneficial or detrimental in the culture of nucleus pulposus cells.

The jelly-like nucleus pulposus (notochord) in the middle of the disc is composed of proteoglycan and functions to disperse the normal loading forces experienced by the spine, acting as a shock absorber to maintain the trunk. However, changes in proteoglycan concentration during age-related disc degeneration are of critical importance. During embryogenesis of the intervertebral disc cells, the cells of the notochord play a critical role in initiating tissue formation and may be directly responsible for development of the nucleus pulposus. In some species, including humans, notochordal cells may eventually be lost and are replaced by chondrocyte-like cells [14,15]. By the age of 60 to 80 years, the intervertebral disc is composed entirely of fiber [16,17]. Accordingly, aging is another risk factor for intervertebral disc degeneration. During degenerative disc disease, loss of disc cells, limited proteoglycan synthesis, and a shift toward synthesis of a fibrotic matrix occur. *Klotho*, a newly identified antiaging gene, has attracted recent attention. The *Klotho *gene encodes a single-pass transmembrane protein. *Klotho *is predominantly expressed in the kidney, but it is also expressed in the brain, parathyroid gland, and heart of normal subjects [18-20]. The *Klotho *gene plays a critical role in regulating aging and in the development of age-related diseases in mammals. Loss of *Klotho *can result in multiple aging-like phenotypes [21,22], and conversely, the overexpression of *Klotho *in *Klotho*^-/- ^mice extends their life span [23]. *Klotho *gene polymorphisms in humans are associated with pathophysiologic bone loss with aging [24], spondylosis [25], osteocalcin levels [26], and bone mineral density [27]. However, no reports exist of the expression of Klotho protein in the intervertebral disc. Moreover, factors responsible for age-associated impairment of intervertebral disc are poorly understood.

In the previous study, we focused on assessing the relation among oxygen tension, klotho, and Wnt signaling and attempted to examine the biologic mechanisms (upstream or downstream) of Wnt signaling in nucleus pulposus cells. The Wnt-signal downstream promoter may be induced by oxygen tension or an age-related gene. Therefore, the purpose of the present investigation was threefold: to determine how Wnt signaling in nucleus pulposus cells is regulated by oxygen tension; to determine the effects of the aging-suppressor gene *Klotho *on Wnt signaling; and to investigate the relation between disc degeneration (maintenance of cell proliferation and matrix synthesis) and *Klotho *expression. These findings provide new insight into the regulation and maintenance of cell proliferation and matrix synthesis by *Klotho *and Wnt signaling cross-talk in nucleus pulposus cells.

## Materials and methods

All animal experiments were performed with approval from Tokai University animal study institutional review board.

### Reagents and plasmids

Plasmids were kindly provided by Dr. Raymond Poon (Hospital for Sick Children, University of Toronto, Toronto, Ontario, Canada) (plasmid encoding wild-type (WT) β-catenin and the backbone), Ilsa Rovira (Cardiology Branch, National Heart, Lung, and Blood Institute, NIH, Bethesda, MD, USA) (plasmid encoding Flag-tagged full-length Klotho) [28], Dr. Young Han Lee (Institute of Biomedical Science and Technology, Konkuk University Hospital, Seoul, South Korea) (plasmids encoding luciferase-tagged Klotho constructs, Del-1, Del-2, Del-3, and Del-4) [29], Dr. Michael C. Naski (University of Texas Health Science Center at San Antonio, San Antonio, TX, USA) (plasmid encoding luciferase-tagged aggrecan) [30], Dr. Sean A. McCarthy (University of Michigan, Ann Arbor, MI, USA) (plasmid encoding Dickkopf-1 (Dkk1), Dkk2, Dkk3, and Dkk4 expression vectors or backbone vector), and Dr. Yoshihiko Yamada (Laboratory of Developmental Biology and Anomalies, National Institute of Dental Research, Bethesda, MD, USA) (plasmid encoding luciferase-tagged type-II collagen) [31]. The constitutively active (CA)-GSK3β (no. 14754) and dominant-negative (DN) GSK3β (no. 14755) were purchased from Addgene (Cambridge, MA, USA). Topflash (optimal Tcf-binding site) and Fopflash (a promoter with a mutated Tcf-binding site) were purchased from Upstate Biotechnology, Inc. (Lake Placid, NY, USA). β-Catenin small interfering RNA (siRNA) (no. sc-29209) and control siRNA duplexes were purchased from Santa Cruz Biotechnology (Santa Cruz, CA, USA). In the Klotho constructs, the TCF/LEF binding motifs within the klotho promoter were analyzed by using the Web-based tool for predicting transcription factor-binding sites in DNA sequences, Transcription Element Search System (TESS). As an internal transfection control, we used the empty vector pGL4.74 (Promega, Fitchburg, WI, USA) containing the *Renilla reniformis *luciferase genes. The amount of transfected plasmid, the pretransfection period after seeding, and the posttransfection period before harvesting were optimized for rat nucleus pulposus cells by using the pSVβ-galactosidase plasmid (Promega). We used 6-bromoindirubin-3'-oxime (BIO) (no. 361550; Calbiochem, San Diego, CA, USA) to examine the activity of the Wnt signaling activity. BIO is a cell-permeable, highly potent, selective, reversible, and ATP-competitive specific inhibitor of GSK-3α/β activity [32]. Recombinant Klotho protein (α-Klotho) (no. 100-53) was purchased from Peprotech (Rocky Hill, NJ, USA).

### Isolation of intervertebral disc cells

In total, 64 (32 female and 32 male) 12-week-old Sprague-Dawley rats were used for this study. None of the variables differed between females and males. Nucleus pulposus cells were isolated by using methods reported by Hiyama *et al.*, 2007 [1]. In brief, the rats were euthanized by injection of an excess of pentobarbital sodium (100 mg/kg) (Nembutal; Abbott Laboratories, Abbott Park, IL, USA). The spinal column was removed under aseptic conditions, and the lumbar intervertebral discs were separated under microscopy. The gel-like nucleus pulposus was separated from the annulus fibrosus. The obtained nucleus pulposus tissue was digested in a mixture of 0.01% trypsin and allowed to digest at 37°C for 15 minutes. The digested tissue was passed through a cell strainer (BD Biosciences, San Jose, CA, USA) with a pore size of 100 μm and was washed twice with phosphate-buffered saline (PBS; Gibco, Invitrogen, Carlsbad, CA, USA). The isolated cells were maintained in Dulbecco modified Eagle medium (DMEM; Gibco, Invitrogen) and 10% fetal bovine serum (FBS; Gibco, Invitrogen) supplemented with antibiotics (1% penicillin/streptomycin) at 37°C in a humidified atmosphere of 5% CO_2_. When confluent, the nucleus pulposus cells were harvested and subcultured in 10-cm dishes. Cells were then counted and plated at the appropriate density. Because cells obtained from the rat intervertebral disc tissues were variable in morphology until passage 2 to 3, we used low-passage (< 3) cells cultured in monolayers for all experiments.

### Cell culture in hypoxia

To induce hypoxia, nucleus pulposus cells were cultured in a mixture of 2% O_2_, 5% CO_2_, and 93% N_2 _for 24 hours. The plating density chosen was dependent on the requirements of the individual assays. The concentration of oxygen and the duration of the incubation period chosen for this study were based on the *in vitro *studies of Risbud *et al. *[33] and on information generated on the oxemic status of the disc *in vivo*.

### Immunofluorescence staining

Nucleus pulposus cells were plated in flat-bottom 96-well plates (5 × 10^3 ^cells/well) and maintained in conditions of normoxia or hypoxia for 24 hours. After this treatment, cells were fixed with 4% paraformaldehyde, permeabilized with 0.2% Triton X-100 (vol/vol) in PBS for 10 minutes, blocked with PBS containing 5% FBS, and incubated overnight at 4°C with antibodies against β-catenin (1:200 dilution; Cell Signaling Technology, Danvers, MA, USA). After washing, the cells were incubated with anti-rabbit IgG Alexa Fluor 594 (red) and/or anti-goat Alexa Fluor 488 secondary (green) antibodies (Invitrogen) at a dilution of 1:50 and 10 μM 4',6-diamidino-2-phenylindole (DAPI) for 1 hour at room temperature for nuclear staining. The samples were observed with a fluorescence microscope connected to a digital imaging system. Similar experiments were performed to assess the expression of anti-rabbit-Klotho (1:200 dilution; Abcam, Cambridge, UK) and anti-goat-Klotho (1:50 dilution; Santa Cruz Biotechnology). Negative controls without the primary antibody were prepared.

### Immunohistologic studies

Freshly isolated rat spines were immediately fixed in 4% paraformaldehyde in PBS, decalcified, embedded in paraffin, sectioned, and assessed by histology and then immunostaining. Sagittal sections were deparaffinized in xylene, rehydrated through a graded ethanol series, and stained with hematoxylin. To localize Klotho, sections were incubated with anti-Klotho antibody (ab75023; Abcam) in 2% bovine serum albumin (BSA) in PBS at a dilution of 1:200 at 4°C overnight. After thoroughly washing the sections, the bound primary antibody was detected with a biotinylated universal secondary antibody (Vector Laboratories, Burlingame, CA, USA) at a dilution of 1:20 for 10 minutes at room temperature. Sections were incubated with a streptavidin/peroxidase complex for 5 minutes and washed with PBS, and the color was developed by using 3'-3-diaminobenzidine (DAB) (VECTASTAIN Universal Quick Kit; Vector Laboratories) and peroxidase DAB substrate kit (Vector), according to the manufacturer's protocol. Negative controls without the primary antibody (anti-Klotho) were prepared.

### 3-(4,5-Dimethylthiazol-2-yl)-2,5-diphenyltetrazolium bromide (MTT) assay

To measure disc-cell proliferation, a modified MTT assay was carried out as previously described [26]. In brief, exponentially growing nucleus pulposus cells were seeded in 24-well plates at 1.5 × 10^4 ^cells/well. After treatment with Klotho (100 ng/ml), MTT diluted in serum-free DMEM was added to the culture medium to a final concentration of 0.5 mg/ml. No data showed the physiological protein concentration of Klotho in the nucleus pulposus cells. We therefore chose to use the concentration of Klotho protein (100 ng/ml) referred to in a previous study [34]. At the end of the incubation period (2 hours at 37°C), the medium was removed, and the precipitated formazan crystals were solubilized in dimethyl sulfoxide (DMSO). Product formation was measured by reading the absorbance at 590 nm by using a microplate reader (Pharmacia).

### Staining for senescence-associated β-galactosidase (β-gal)

To stain nucleus pulposus cells for senescence-associated β-gal activity, we used a cell-senescence histochemical staining kit (CS0030; Sigma-Aldrich, St. Louis, MO, USA) according to the previously described protocol [26]. Cells were treated for 24 hours with or without 100 ng/ml Klotho. To quantify the results, a minimum of 100 cells spanning five different microscopy fields were scored for staining, and results represent the mean ± standard deviation (SD).

### Cell-cycle analysis by flow cytometry

Nucleus pulposus cells were grown in 24-well plates under a humidified 5% CO_2 _atmosphere at 37°C. The seeding density was 5 × 10^4 ^cells/ml. The cells were allowed to adhere for 24 hours in medium containing 2% FBS. The culture medium in each flask was then replaced with medium containing 0.5% FBS. Klotho (100 ng/ml) was added to this medium as a concentrated stock solution dissolved in DMSO. After an additional incubation period of 24 or 48 hours, the cell-cycle distribution of the nucleus pulposus cells was analyzed with flow cytometry after DNA staining with propidium iodide by using the CycleTEST PLUS kit (BD Pharmingen, San Diego, CA, USA). CELLQuest (BD Pharmingen) and ModFit LT (BD Pharmingen) software packages were used for cell acquisition and analysis. Each plot represents the analysis of 10,000 events. The histograms present typical results, and the percentages of cells in G_1_, S, and G_2_/M cell-cycle phases are shown as the means of triplicate measurements. The results from three individual experiments are shown.

### Total RNA extraction and real-time reverse transcriptase (RT)-PCR analysis

Total RNA was extracted from rat nucleus pulposus and annulus fibrosus cells or tissues by using the TRIzol RNA isolation protocol (Invitrogen). Before elution from the column, RNA was treated with RNase-free DNAse I. Total RNA (100 ng) was used as a template for the real-time PCR analyses. The cDNA was synthesized by the reverse transcription of mRNA by using the protocol described previously (26). The real-time PCR analyses were performed in triplicate by using 96-well plates with the Fast SYBR Green Master Mix (Applied Biosystems, Carlsbad, CA, USA). Two microliters of cDNA per sample was used as the template for real-time PCR: 1 μl of forward primer and 1 μl of reverse primer were added to 20 μl SYBR Green Master Mix. PCR reactions were performed in an Applied Biosystems 7500 Fast system, according to the manufacturer's instructions. All primers for *β-catenin*, *Klotho*, and *aggrecan *were synthesized by Takara Bio, Inc. (Tokyo, Japan): *β-catenin *(NCBI number: AF_121265.1): forward, 5'-GCCAGTGGATTCCGTACTGT-3' and reverse, 5'-GAGCTTGCTTTCCTGATTGC-3'; *Klotho *(NCBI number: NM_031336.1): forward, 5'-CGATGTTCGTGACAGCCAATG-3' and reverse, 5'-GTTGATGCCGTCCAACACGTAG-3'; and *aggrecan *(NCBI number: NM_022190.1): forward, 5'-TCCGCTGGTCTGATGGACAC-3' and reverse, 5'-CCAGATCATCACTACGCAGTCCTC-3'. To normalize each sample, a control gene (GAPDH) was used, and the arbitrary intensity threshold (C_t_) of amplification was computed. The expression scores were obtained by the ΔΔC_t _calculation method.

### Gene-suppression studies by small interfering RNA (siRNA)

We silenced β-catenin expression in nucleus pulposus cells by using small interfering RNA (siRNA) technology. In brief, nucleus pulposus cells were transferred to 24-well plates at a density of 6 × 10^4 ^cells/well 1 day before transfection. The next day, cells were treated with β-catenin siRNA or control siRNA duplexes at a final concentration of 100 to 500 ng by using Lipofectamine 2000. Cells also received Klotho promoter constructs and the pGL4.74 plasmid at the time of transfection. Six hours after transfection, the medium was replaced with complete growth medium, and the cells were allowed to recover for 18 hours. Cells were then cultured for 24 hours, and luciferase activity was measured.

### Methylation analysis

Genomic DNA was extracted from cells not treated with BIO and BIO-treated cells by using a FastPure DNA Kit (Takara Bio), and bisulfite treatment of genomic DNA was performed by using MethylEasy Xceed (Human Genetic Signatures) and following the manufacturers' instructions. The bisulfite-treated DNA was amplified by PCR with Takara *Taq *Hot Start Version (Takara Bio) and T-Vector pMD20 (Takara Bio) for cytosine-phosphate-guanosine (CpG)-rich regions around the rat *Klotho *gene. PCR products amplified by using Takara LA Taq HS were subcloned into T-Vector pMD20, and sequence analysis was performed. All methylation studies were performed by bisulfite modification of DNA, which converts all unmethylated CpG sites to uracil-phosphate-guanosine, leaving methylated CpGs intact. The DNA was then amplified with the following gene-specific primers: *Klotho*, forward, 5'-TGATGTGGGGATATTTTAGGA-3', and reverse, 5'-CAACAAATACAACRACAACAAA-3'. After sequencing of the amplified DNA, methylated CpGs were identified with visual inspection of the sequencing traces in the electropherograms and by comparison with sequencing traces of BIO-treated cell gene sequences relative to the reference in the control cell sequence.

### Western blotting analysis

After treatment, nucleus pulposus cells were immediately washed 3 times with ice-cold PBS and solubilized in lysis buffer. Proteins were prepared by using the CellLytic NuCLEAR extraction kit (Sigma-Aldrich). All wash buffers and the final resuspension buffer included × 1 protease inhibitor cocktail (Roche, Switzerland), NaF (5 m*M*), and Na_3_VO_4 _(200 μ*M*). Total lysates (30 to 60 μg of protein/lane) were subjected to electrophoresis by using 10% sodium dodecylsulfate-polyacrylamide gel electrophoresis (SDS-PAGE) (Bio-Rad, Hercules, CA, USA). The resolved proteins were transferred electrophoretically to nitrocellulose membrane "blots". The blots were blocked with 5% BSA in TBST (50 m*M *Tris, pH 7.6, 150 m*M *NaCl, 0.1% Tween 20) and incubated overnight at 4°C in 5% BSA in TBST with anti-β-catenin (1:1,000; Cell Signaling) or anti-Klotho (1:1,000; Abcam). Immunolabeling was detected with electrochemiluminescence reagent (Amersham Biosciences, Piscataway, NJ, USA). Quantification of Western blots was performed by using Image J pixel analysis (NIH Image software). Data from Western blots are presented as band density normalized to the loading control (actin).

### Transfections and dual luciferase assay

Nucleus pulposus cells were transferred to 24-well plates at a density of 6 × 10^4 ^cells/well 1 day before transfection. In several experiments, cells were cotransfected with 100 to 500 ng of expression plasmids or the backbone vector, along with reporter plasmids. Lipofectamine 2000 (Invitrogen) was used as a transfection reagent. After 24 hours, cells were maintained in either the hypoxic or the normoxic condition. Twenty-four hours after treatment, the cells were harvested, and a Dual-Luciferase reporter assay system (Promega) was used for the sequential measurements of firefly and *Renilla *luciferase activities. The results were normalized for transfection efficiency and are expressed as a relative ratio of luciferase to pGL4.74 activities (denoted as relative activity). To check the transfection efficiency, nucleus pulposus cells were transfected with plasmid encoding green fluorescent protein; the transfection efficiency for nucleus pulposus cells was 60% to 70%. In addition, the overexpression or silencing efficiency of the targets was confirmed at the mRNA level or protein level. The luciferase activities and relative ratios were quantified by using a Turner Designs Luminometer Model TD-20/20 instrument (Promega).

### Statistical analysis

Typically, data were compiled from at least three independent triplicate experiments, each performed on separate cultures and on separate occasions. We calculated and have displayed the responses as fold change (over the untreated control). Data are presented as mean ± SD. Comparisons of data between groups were performed by using the Student *t *test or ANOVA for assessing variance. Statistical significance (*P *< 0.05) is denoted with an asterisk.

## Results

### Oxygen tension directly regulates Wnt signaling in nucleus pulposus cells

**Figure 1:**
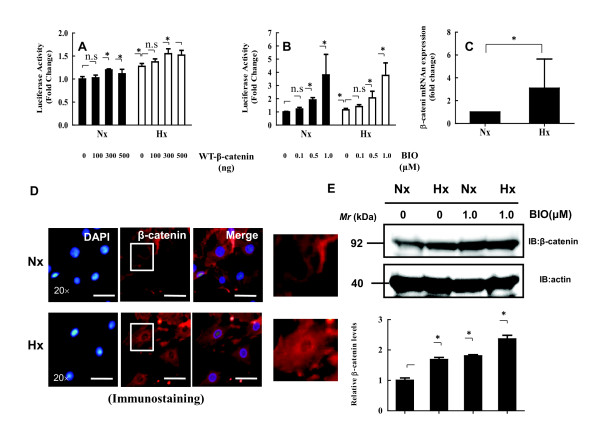
**The effect of oxygen tension on Wnt signaling in nucleus pulposus cells**. **(A) **Nucleus pulposus cells were cotransfected with Topflash (400 ng) and increasing concentrations of the WT β-catenin expression plasmid (100 to 500 ng) under normoxic or hypoxic conditions. **(B) **Topflash was transfected with the pGL4.74 vector into nucleus pulposus cells, and cells were stimulated with BIO (0.1, 0.5, or 1.0 μ*M*) for 24 hours under normoxic or hypoxic conditions. **(C) **Real-time reverse transcription-polymerase chain reaction analysis of β-catenin mRNA levels under normoxic or hypoxic conditions for 24 hours in nucleus pulposus cells. Data are presented as mean ± SD (*n *= 6). *P < 0.05 between groups (A to C). **(D) **Detection of β-catenin expression with immunofluorescence microscopy. Nucleus pulposus cells were cultured under normoxic or hypoxic conditions for 24 hours, fixed, and stained with an antibody against β-catenin. Left: Cells stained with DAPI to identify healthy nuclei. Middle: Cells stained with an antibody to β-catenin. Right: Cells stained with an antibody to β-catenin and DAPI. Scale bar, 50 μm (original magnification, × 20). Boxed areas in the middle panel are magnified in the two panels to the far right. **(E) **A representative Western blot showing an increase in β-catenin protein levels, detectable after 24 hours under the hypoxic condition. kDa, protein-size marker in kilodaltons. Relative quantitation of β-catenin protein levels with Western blot analysis was done by using the Image J software.

We first examined whether oxygen tension activates the transcriptional activity of the Wnt signal pathway by using the TCF- and β-catenin-dependent Topflash reporter plasmid in nucleus pulposus cells. Figure 1A and 1B shows that under the hypoxic condition, Topflash activity increased significantly by 1.3- to 1.5-fold in nucleus pulposus cells after 24 hours of incubation (*P *< 0.05). Nucleus pulposus cells cotransfected with the WT-β-catenin expression plasmid, or treated with a different concentration of BIO, showed a dose-dependent increase in the activity of Topflash. Fopflash promoter (a promoter with a mutated TCF-binding site) activity was unresponsive to oxygen tension (data not shown). We next measured the relative expression level of β-catenin mRNA under normoxic and hypoxic conditions. We analyzed mRNA expression with real-time RT-PCR. The β-catenin mRNA level was threefold higher in nucleus pulposus cells cultured under the hypoxic (2% O_2_) compared with the normoxic condition (21% O_2_) (*P *< 0.05) (Figure 1C). To confirm the dependence of β-catenin expression on oxygen tension, as shown in Figure 1D, we showed that total β-catenin level and the nuclear translocation of β-catenin increased to a greater extent in nucleus pulposus cells cultured under the hypoxic condition than in cells cultured under the normoxic condition. In the hypoxic environment, a significant increase in the levels of β-catenin protein was seen (Figure 1D; arrows). To explore the premise that oxygen tension regulates β-catenin protein expression, nucleus pulposus cells were treated with BIO under normoxic and hypoxic conditions. The expression of β-catenin protein in nucleus pulposus cells was induced by hypoxia (Figure 1E).

### Expression of Klotho by intervertebral disc

**Figure 2:**
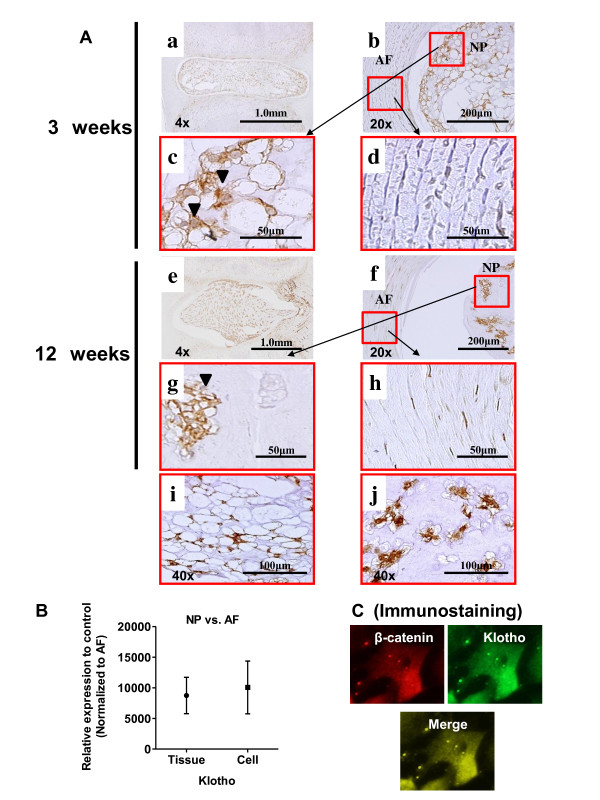
**Expression profiles of Klotho in intervertebral discs. (A) **Sagittal sections from 3-week-old (a-d, i) and 12-week-old (e-h, j) rats. Sections were treated with anti-Klotho antibody and counterstained with hematoxylin. Note that nucleus pulposus cells expressed Klotho protein (arrows in d and h). A magnified view of the areas (panel b and f) is marked by the square in panels c, d, g, and h. **(B) **Real-time RT-PCR analysis of Klotho mRNA levels in nucleus pulposus (NP) and annulus fibrosus (AF). Scale bar, 50 μm to 1.0 mm (original magnification, × 4, × 20, × 40). **(C) **Immunofluorescence was performed after treatment of BIO in nucleus pulposus cells. The subcellular distribution of β-catenin is shown in red; Klotho, in green; and degree of merge, in yellow.

To examine whether Klotho is involved in intervertebral disc differentiation, we first determined the expression profiles of Klotho in intervertebral discs. Expression of the Klotho protein and mRNA was analyzed by immunostaining and real-time PCR. We immunostained discs in sagittal sections from 3-week-old (Figure 2A, a-d, i) and 12-week-old (Figure 2A, e-h, j) rats by using an antibody to Klotho. Klotho was expressed in cells of the nucleus pulposus (Figure 2A, d), but not of the annulus fibrosus (Figure 2A, c), in the 3-week-old rat discs. Klotho expression was higher in the annulus fibrosus of discs in 12-week-old rats compared with 3-week-old rats. The nucleus pulposus of 3-week-old rats (Figure 2A, i) was full of notochordal cells with large vacuoles, and much of the staining was nuclear, which contrasted with the appearance in samples from 12-week-old rats (Figure 2A, j). To determine the expression of Klotho in intervertebral discs, we also measured Klotho mRNA expression both in tissue or cells and found that it was significantly higher in nucleus pulposus than in annulus fibrosus (Figure 2B). We sought to determine whether Klotho and the Wnt signal could form a direct molecular complex in nucleus pulposus cells. We demonstrated that the subcellular distribution of Klotho and β-catenin proteins within BIO-treated cells overlapped (Figure 2C).

### Decreased Klotho transcriptional activity in nucleus pulposus cells treated with hypoxia

**Figure 3:**
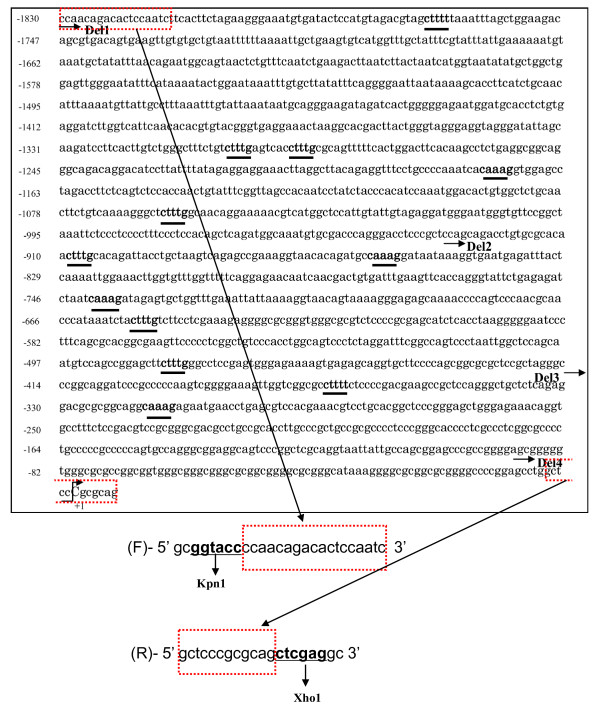
**TCF- and LEF-binding motifs contained in the *Klotho *promoter**. The DNA sequence of the promoter region of the *Klotho *gene. TCF/LEF (CTTTT, CTTTG, or CAAAG) consensus sequences are marked in **bold **type and underlined. The arrows indicate the starting location of the primers used to generate the promoter constructs. The transcription start site is marked as +1; CGC marks the translation start site. The PCR products were digested with *Kpn*I and *Xho*I, and ligated into the *Kpn*I and *Xho*I sites of the pGL3-basic vector [29].

**Figure 4:**
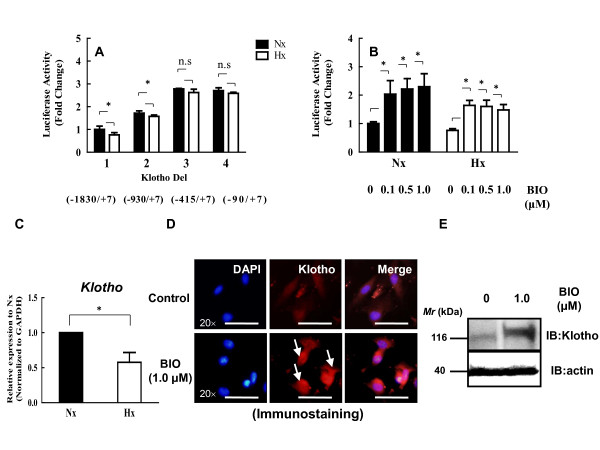
**Effect of oxygen tension and Wnt signaling on *Klotho *gene transcription and protein expression in nucleus pulposus cells**. **(A) **Basal activities of the Klotho reporter constructs in nucleus pulposus cells under normoxic or hypoxic conditions were measured with dual luciferase assay. **(B) **The effect of BIO on Klotho promoter activity in nucleus pulposus cells under normoxic or hypoxic conditions was measured. Data are presented as mean ± SD. *P < 0.05. ns, not significant (A, B). **(C) **Real-time RT-PCR analysis of Klotho mRNA levels under normoxic or hypoxic conditions in nucleus pulposus cells. Data are presented as mean ± SD (*n *= 6). *P < 0.05 between groups. **(D) **Detection of Klotho protein expression by immunofluorescence microscopy. Nucleus pulposus cells were cultured with or without 1.0 μ*M *BIO for 24 hours, fixed, and stained with an antibody against Klotho. Left: Cells stained with DAPI to identify healthy nuclei. Middle: Cells stained with antibody to Klotho. Right: Cells stained with antibody to Klotho and DAPI. Scale bar, 50 μm (original magnification × 20). **(E) **Western blot analysis detected an increased amount of Klotho protein after treatment with BIO for 24 hours.

We first investigated the regulation of Klotho expression to determine whether a relation existed between Klotho and the oxemic status of the nucleus pulposus cell by analyzing the 1.83-kb promoter sequence of human Klotho and measuring the activities of different sizes of promoter constructs (Figure 3). Sequence analysis shows that the Klotho promoter contains 12 TCF/LEF-binding motifs (CTTTT, CTTTG, or CAAAG); that is, 12 TCF/LEF-binding motifs in the p-Klotho Del1 (-1830/+7) construct, but no TCF/LEF-binding motifs in the p-Klotho Del4 (-90/+7) construct. To analyze the promoter function further, we used luciferase reporter constructs containing -1830/+7 bp (p-Klotho-Del1), -930/+7 bp (p-Klotho-Del2), -415/+7 bp (p-Klotho-Del3), or -90/+7 bp (p-Klotho-Del4) constructs of the Klotho promoter [29]. We measured the basal activity of all four constructs in nucleus pulposus cells. Figure 4A shows that the p-Klotho-Del3 construct had maximal basal activity, and the p-Klotho-Del1 construct exhibited the lowest activity. We next examined the effect of oxygen tension on all Klotho promoter activities. Experiments using serial deletion constructs of the Klotho promoter under the hypoxic condition showed a significant decrease in the activity of the p-Klotho-Del1 and p-Klotho-Del2 promoters but not of the p-Klotho-Del3 and p-Klotho-Del4 promoters (Del1: normoxic 1.00 ± 0.15, hypoxic 0.71 ± 0.10; Del2: normoxic 1.71 ± 0.10, hypoxic 1.53 ± 0.01; Del3: normoxic 2.78 ± 0.02, hypoxic 2.62 ± 0.14; Del4: normoxic 2.59 ± 0.05, hypoxic 2.71 ± 0.12) (Figure 4A). To evaluate these results further, a p-Klotho-Del1 reporter plasmid (Klotho-luc) was transfected along with the pGL4.74 vector into nucleus pulposus cells, and the cells were stimulated with BIO under the normoxic and hypoxic conditions. Figure 4B shows that the Klotho promoter activity was increased by BIO treatment. Cells cultured under the hypoxic condition showed lower Klotho promoter activity compared with the normoxic condition. Real-time RT-PCR analysis also showed that hypoxia decreased the expression of Klotho mRNA to 0.5 to 0.7 to that in normoxia (Figure 4C). To determine whether a concomitant elevation in Klotho protein expression was associated with Wnt signaling, the cells were evaluated with immunofluorescence analysis (Figure 4D) and Western blotting (Figure 4E). As shown in Figure 4D, immunofluorescence analysis with an anti-Klotho antibody showed that BIO treatment (1.0 μ*M*, 24 hours) promoted the nuclear translocation of Klotho more strongly in nucleus pulposus cells than in untreated cells. Klotho was localized to the nucleus in cells treated with BIO (arrows). Similarly, immunoblotting analysis of nucleus pulposus cells treated with BIO (1.0 μ*M*, 24 hours) showed more Klotho protein than in control nucleus pulposus cells (Figure 4D).

### Regulation of Klotho by Wnt signaling in nucleus pulposus cells

**Figure 5:**
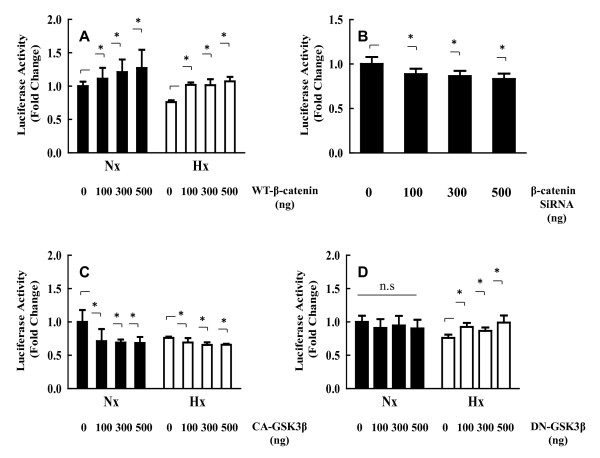
**Effect of normoxic and hypoxic conditions on *Klotho *promoter activity**. Nucleus pulposus cells were cotransfected with the Klotho reporter plasmid along with WT-β-catenin **(A)**, β-catenin siRNA (Si-β-catenin) **(B)**, constitutively active (CA)-GSK3β **(C)**, dominant-negative (DN)-GSK3β **(D)**, or empty vectors and the pGL4.74 vector. Cells were cultured under normoxic or hypoxic conditions for 24 hours, and luciferase reporter activity was measured. The results were normalized for transfection efficiency and are expressed as a relative ratio of luciferase to pGL4.74 activity (denoted as relative activity). Data are presented as mean ± SD. *P < 0.05. ns, not significant.

Although Wnt signaling has also been shown to have a role in nucleus pulposus cells, interactions between Wnt signaling and Klotho in nucleus pulposus cells have not been previously described. Initial experiments were performed to investigate the role of Wnt signaling in the transcriptional activity of Klotho in nucleus pulposus cells. Nucleus pulposus cells were transiently transfected with plasmids encoding Klotho along with WT-β-catenin expression plasmid. Figure 5A shows that forced expression of β-catenin under both normoxic and hypoxic conditions significantly induced Klotho promoter activity (*P *< 0.05). To validate these findings, we performed loss-of-function experiments by using siRNA for β-catenin. Suppression of gene expression was confirmed by real-time RT-PCR (data not shown). Under the normoxic condition, Klotho promoter activity was inhibited in nucleus pulposus cells cotransfected with β-catenin siRNA (*P *< 0.05; 100 ng, *P *= 0.005; 300 ng, *P *= 0.002; 500 ng, *P *= 0.0001) (Figure 5B). We also examined the effect of GSK3β treatment on Klotho promoter activity. Nucleus pulposus cells were transiently cotransfected with plasmids encoding CA-GSK3β (Figure 5C) or DN-GSK3β (Figure 5D). Klotho promoter activity was decreased in a dose-dependent manner by the CA-GSK3β expression plasmid (*P *< 0.05) but not by the DN-GSK3β expression plasmid. These results suggest that Klotho is downstream of β-catenin signaling in nucleus pulposus cells. To confirm whether Dkk, a secretory protein that inhibits Wnt signaling by disrupting the interactions between Wnt and Fz, regulates the transcriptional activity of Klotho, we assessed the transcriptional activity of Klotho after treatment with Dkk1, 2, 3, or 4 (500 ng) expression plasmids or backbone vector for 24 hours. As shown in Additional file 1 Klotho activity was not affected by Dkk treatment.

### Klotho suppression of the proliferation of nucleus pulposus cells

**Figure 6:**
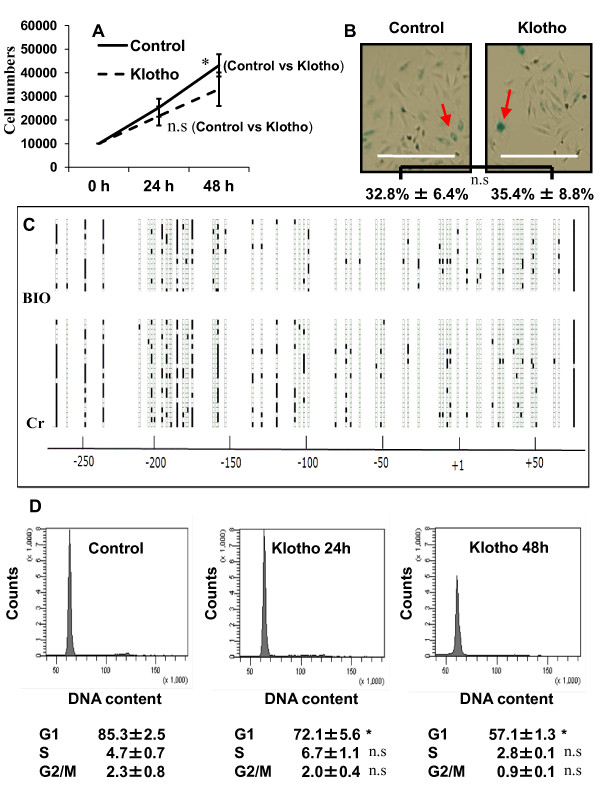
**Regulation of cell proliferation by Klotho**. **(A) **Nucleus pulposus cells were treated with Klotho (100 ng/ml) in 96-well plates. Cell proliferation was evaluated by using the MTT viability assay, as described in Materials and methods. Data are presented as mean ± SD (*n *= 12). *P < 0.05 between groups; ns, not significant. **(B) **Photomicrographs showing staining of nucleus pulposus cells for senescence-associated β-gal determined as the percentage of positive cells. Positive staining for senescence-associated β-gal was detectable for 24 hours after Klotho treatment. Bar, 500 μm (original magnification × 10). **(C) **BGS analysis of the Klotho promoter region in BIO-treated cells (BIO) and untreated cells (Cr). Each row of circles represents the DNA sequence of individual clones. The open and solid circles represent the unmethylated and methylated CpG sites, respectively. **(D) **Nucleus pulposus cells were cultured for 24 hours, the cells were incubated with or without Klotho (100 ng/ml) for 24 to 48 hours and harvested, and the nuclei were stained with propidium iodide. DNA histograms were generated by using flow cytometry. *P < 0.05 between groups; ns, not significant.

Proliferation of nucleus pulposus cells was decreased significantly by Klotho at 48 hours but not 24 hours after the treatment (*P *< 0.05 and *P *= 0.24, respectively) (Figure 6A). To test for cell senescence, we stained cells for the expression of the senescence-associated protein, β-gal. As shown in Figure 6B, Klotho treatment for 24 hours did not change the percentage of senescence-associated β-gal-positive nucleus pulposus cells (arrow in control, 32.8% ± 6.4%; Klotho, 35.4% ± 8.8%). In addition, Klotho exposure did not increase the level of apoptosis (data not shown). To analyze the methylation status of the CpG islands in the Klotho promoter region, bisulfite genomic sequencing (BGS) analysis was performed in BIO-treated nucleus pulposus cells. As shown in Figure 6C, a dense GC-rich region was identified around the -200 bp site of Klotho. To determine the methylation density of the Klotho promoter region, which encompasses 61 CpGs, we performed BGS analysis of BIO-treated and untreated nucleus pulposus cells. The methylation of the Klotho promoter region was much less extensive in the BIO-treated cells with activated Wnt signaling (that is, activation of Wnt signaling tended to be associated with unmethylated CpG islands). These data strongly suggest that the transcriptional activation of Klotho is associated with aberrant promoter demethylation by Wnt signaling in nucleus pulposus cells.

With flow cytometry, we evaluated whether the cell-cycle status was affected by Klotho (100 ng) treatment at different times after treatment. The number of cells in the G_1 _phase was significantly decreased in the presence of Klotho compared with untreated cells (24 hours, *P *= 0.035; 48 hours, *P *< 0.05). These results showed that activation of Klotho can suppress the proliferation of nucleus pulposus cells and may induce cells to enter a resting phase, the G_0 _phase (Figure 6D).

### Functional role of Klotho and Wnt signaling crosstalk in nucleus pulposus

**Figure 7:**
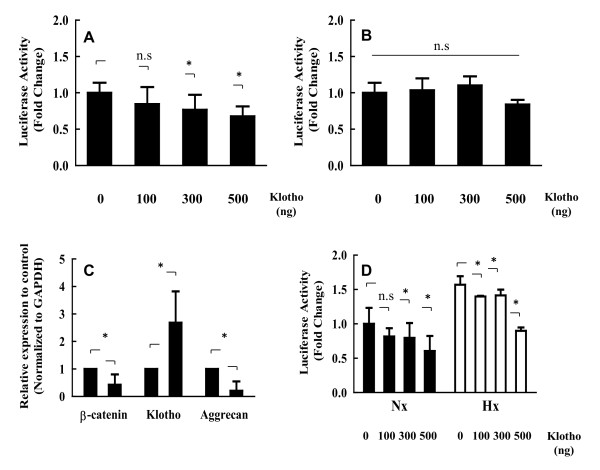
**Regulation of aggrecan and Wnt signal expression by Klotho**. **(A**, **B) **To define the functional relevance of Klotho in nucleus pulposus cells, nucleus pulposus cells were cotransfected with the aggrecan reporter plasmid (Agg-luc) **(A)** or the collagen II reporter plasmid (Col2-luc) **(B) **along with the Klotho expression plasmid or empty vectors and the pGL4.74 vector, and the activities of the reporter were measured by using a dual luciferase assay. Data are presented as mean ± SD. *P < 0.05. ns, not significant. **(C) **Real-time RT-PCR analysis of β-catenin, Klotho, or aggrecan mRNA levels in nucleus pulposus cells treated with Klotho (500 ng/ml). Data are presented as mean ± SD (*n *= 6). *P <0.05 between groups. **(D) **Nucleus pulposus cells were cotransfected with Topflash (400 ng) and increasing concentrations of the Klotho expression plasmid (100 to 500 ng) under normoxic or hypoxic conditions. Data are presented as mean ± SD. *P < 0.05.

To investigate the possible mechanisms underlying the actions of Klotho in the induction of nucleus pulposus cell markers, we next examined the effect of Klotho treatment on expression of aggrecan (Figure 7A) and collagen type II (Figure 7B) under the normoxic condition. Cotransfection of nucleus pulposus cells with a Klotho expression plasmid significantly decreased aggrecan reporter activity (Klotho 100 ng, *P *= 0.085; Klotho 300 ng, *P *= 0.011; Klotho 500 ng, *P *= 0.001) but not collagen type-II reporter activity (Klotho 100 ng, *P *= 0.723; Klotho 300 ng, *P *= 0.308; Klotho 500 ng, *P *= 0.126). To confirm the regulatory role of Klotho in the expression of aggrecan and collagen type II, we performed real-time PCR to examine changes in the expression of aggrecan and collagen type II in nucleus pulposus cells. The results showed that treatment with 500 ng of Klotho for 24 hours also decreased aggrecan mRNA levels in nucleus pulposus cells, as shown by real-time PCR (*P *< 0.05; *P *= 0.004) (data not shown for collagen type II) (Figure 7C). In addition, we used immunofluorescence analysis and Western blotting to confirm the role of Klotho in aggrecan protein expression. The results demonstrated that Klotho treatment (100 ng/ml, 24 hours) decreased aggrecan protein levels to a greater extent in nucleus pulposus cells than in control cells (data not shown). Finally, to confirm the relation between Wnt signaling and Klotho and to assess whether Klotho inhibits Wnt signaling in nucleus pulposus cells, we used a Klotho expression plasmid in the Topflash reporter assay. Topflash reporter activity was decreased by Klotho treatment in nucleus pulposus cells (normoxic: Klotho 100 ng, *P *= 0.089; Klotho 300 ng, *P *= 0.044; Klotho 500 ng, *P *= 0.0003; hypoxic: Klotho 100 ng, *P *= 0.001; Klotho 300 ng, *P *= 0.002; Klotho 500 ng, *P *= 0.0001) (Figure 7D). Similarly, Klotho treatment inhibited gene expression of β-catenin compared with untreated control cells (*P *< 0.05; *P *= 0.023) (Figure 7C). Real-time PCR analysis also indicated that a concomitant increase in Klotho mRNA levels occurred when nucleus pulposus cells were treated with exogenous Klotho (*P *< 0.05; *P *= 0.028) (Figure 7C). These findings indicate that Wnt signaling is inhibited by Klotho in nucleus pulposus cells.

## Discussion

In this study, we focused on Wnt signaling to clarify the mechanism (upstream or downstream) underlying intervertebral disc degeneration. First, we examined the relation between Wnt signaling and the oxemic status of nucleus pulposus cells. Nucleus pulposus is an avascular tissue, and in some species, pO_2 _levels in large discs may be low. Oxygen tension is a powerful stimulus of nucleus pulposus cells, yet the net result of hypoxia--anabolic or catabolic--in nucleus pulposus cells is not known. In addition, the mechanism by which the nucleus pulposus cells survive in the hypoxic environment of the intervertebral disc remains largely unknown, and no evidence suggests how oxygen levels change with disc degeneration *in vivo*. However, *in vitro *studies demonstrate stimulatory effects of hypoxia on nucleus pulposus proliferation and matrix synthesis [35,36]. It is very difficult to conclude that low oxygen would be bad for discs from all of these study data, because the existence of complex signaling networks is found in disc degeneration. However, in this study, we found a hypoxia-dependent increase in the expression of β-catenin and a concomitant increase in Wnt signaling activity. We demonstrated previously that activation of Wnt signaling elevates the gene expression and activity of aggrecan by the balance of catabolic and anabolic factors in nucleus pulposus cells. This is the key to understanding the biology of intervertebral disc maintenance and degeneration [26]. Mwale *et al. *[37] reported that low oxygen levels increase aggrecan mRNA expression in nucleus pulposus cells and that disc cell metabolism is not impaired at low oxygen concentrations. Our results indicate that effect of hypoxia on matrix synthesis of the nucleus pulposus cells might be directly mediated by Wnt signaling or indirectly by Wnt stimulation of other pathways, such as the hypoxia-inducible factor (HIF) pathway. However, this study was unable to distinguish whether this is a direct or an indirect effect. Kaidi *et al. *[38] reported that Wnt signaling is mediated by the physical interaction between HIF-1α and β-catenin. Intriguingly, the β-catenin/HIF-1α interaction increases HIF transcriptional activity, which might help cells adapt to severe hypoxia. Risbud *et al. *[39] reported that nucleus pulposus cells display robust and constitutive expression of both HIF-1α and HIF-2α, suggesting that these cells reside in a hypoxic environment. Future studies will address the role of the β-catenin/HIF pathways to determine whether regulation of Wnt signaling by hypoxic conditions is specific to nucleus pulposus cells.

Second, we investigated the expression of Klotho, a newly identified antiaging gene, and examined the relation in nucleus pulposus cells of Wnt signaling and Klotho.

Finally, we investigated the role of Klotho on aggrecan or collagen type-II expression and the proliferation of nucleus pulposus cells.

It is clear that the incidence of disc degeneration increases markedly with age. Several studies have focused on Klotho and its role in the aging phenotype. The Klotho protein is composed of a large extracellular domain (130 kDa), a transmembrane domain, and a very short intracellular domain (10 amino acids). The entire extracellular domain is released into the extracellular space and is detectable in blood, urine, and cerebrospinal fluid [40]. Membrane Klotho functions as a receptor for a hormone that regulates excretion of phosphate and synthesis of active vitamin D in the kidney [41,42]. Secreted Klotho regulates the activity of multiple growth factors, including insulin/insulin-like growth factor-1 (IGF-1) [11], Wnt [28], and transforming growth factor (TGF)-β1 [43]. Liu *et al. *[28] demonstrated that Klotho acts as a Wnt antagonist and suggested that prolonged Wnt stimulation may contribute to stem cell depletion and aging. They reported that secreted Klotho inhibits Wnt signaling activity by binding directly to Wnt ligands and preventing them from binding to their receptors. In this study, we performed molecular functional analysis to study the expression of Klotho in the intervertebral disc and to elucidate the signal crosstalk between Klotho and Wnt signaling; this crosstalk is attracting attention as a potential trigger for degeneration in the intervertebral disc. Interestingly, we demonstrated for the first time that the transcriptional activation of Klotho is associated with promoter demethylation by Wnt signaling. Accordingly, these experiments strongly suggested that in nucleus pulposus cells, activation of Wnt signaling induces an increase in Klotho promoter activity and gene and protein expression by demethylation. By contrast, activation of nucleus pulposus cells by secreted Klotho seems to suppress Wnt signaling. These results might support that Klotho is not simply an antagonist of Wnt signaling but that Wnt signaling and Klotho form a negative-feedback loop in nucleus pulposus cells.

We hypothesized that Wnt signaling might control nucleus pulposus homeostasis in normal intervertebral discs. However, although we know that, in general, Klotho is an antiaging gene, activation of Wnt signaling nevertheless resulted in induction of Klotho in nucleus pulposus cells. Consequently, Klotho inhibited aggrecan synthesis and proliferation of nucleus pulposus cells. This is unexpected, because suppression of aggrecan synthesis and proliferation of nucleus pulposus cells would appear to be potentially damaging to the intervertebral disc. The reason for these discrepant effects is not clear, but several possible explanations exist.

First, it is possible that Klotho interacts with signal pathways other than Wnt signaling. Klotho can affect the function of Wnt, TGF-β1, and IGF, which can affect the expression of aggrecan and collagen type II in different ways. Doi *et al. *[43] reported that secreted Klotho suppresses TGF-β1 signaling by directly binding to type-II TGF-β receptor (TGFβR2) on the cell surface and preventing TGF-β1 binding to that receptor. TGF-β1 is the most potent inducer of matrix synthesis in nucleus pulposus cells. In addition, fibroblast growth factor (FGF) may act as a catabolic and antianabolic mediator in nucleus pulposus cells, stimulating matrix metalloproteinase (MMP)-13 expression and suppressing proteoglycan synthesis. Recently, Kuro-o [44] reported that the most important function of the Klotho family is to regulate the function of the endocrine FGF family. Therefore, the final effect of Klotho on the aggrecan and collagen type II gene expression may be the result of the different effects.

Second, these mechanisms may be specific to the nucleus pulposus cells, because this cell is unique both embryologically and functionally. Moreover, although regulation of Wnt signaling may be tissue specific, the signaling pathway has been shown to modulate cellular proliferation or differentiation.

Third, these differences may relate to the age and species of the animal from which the cells are isolated and the environment in which the cell metabolism was studied. Other groups have shown that glycosaminoglycan production by nucleus pulposus and annulus fibrosus cells can vary, depending on the source of disc cells [45].

Fourth, the *Klotho *gene encodes several isoforms in mammals, which include a membrane-bound and two secreted forms. Therefore, the results for this study might be from the different isoforms through different signaling pathways.

Future studies will address the role of Wnt signaling by using human samples to determine whether the regulation of cell growth and matrix synthesis by Klotho is specific to nucleus pulposus cells. Moreover, it is necessary to examine whether, in nucleus pulposus cells, Klotho affects the activity of several signaling pathways, including those of TGF, FGF, or the MMP family, which may participate in intervertebral disc degeneration. Wnt signals are regulated in various ways in response to various extracellular and intracellular signaling. However, DKK, which regulates extracellular Wnt signaling, did not inhibit the reporter activation of Klotho in this study. The DKK family comprises four subtypes, which affect Wnt signaling by combining with the collaboration receptor LRP5/6 of each Wnt-Fzd [46,47]. The subtypes seem to influence the functional differences and types of cells that develop, although it is generally believed that only DKK1 and DKK4 inhibit Wnt signals. Our present results suggest that the action of the regulatory factor DKK outside of cells is not dependent on the expression of Klotho in nucleus pulposus cells.

## Conclusions

Here, we demonstrated that (1) hypoxia stimulates Wnt signaling in nucleus pulposus cells; (2) Klotho is expressed in intervertebral discs; (3) transcriptional activation of Klotho is associated with promoter demethylation by Wnt signaling; and (4) Wnt signaling and Klotho form a negative-feedback loop in nucleus pulposus cells. In summary, our study demonstrates that in nucleus pulposus cells, the expression of Klotho is regulated by the balance between upregulation and downregulation of Wnt signaling. To elucidate the mechanism causing intervertebral disc degeneration, further studies are needed to determine how the interplay of various Wnt ligands and Klotho modulates cell growth and matrix synthesis in nucleus pulposus cells.

## Abbreviations

BGS: bisulfite genomic sequencing; BIO: 6-bromoindirubin-3'-oxime; CPG: cytosine-phosphate-guanosine; DKK: Dickkopf; HIF-1α: hypoxia-inducible factor 1α; TCF/LEF: T-cell factor/lymphoid enhancer factor.

## Competing interests

The authors declare that they have no competing interests.

## Authors' contributions

AH and FA made substantial contributions to conception and design, or acquisition of data, or analysis and interpretation of data. AH, FA, KY, DS, and JM were involved in drafting the manuscript or revising it critically for important intellectual content. All authors read and approved the manuscript for publication.

## Supplementary Material

Additional file 1 **Nucleus pulposus cells were cotransfected with the Klotho reporter plasmid along with 500 ng of Dkk1, Dkk2, Dkk3, Dkk4, or the empty backbone vector, and the reporter activity was measured (left panel)**. Nucleus pulposus cells were cotransfected with the Klotho promoter plasmid along with increasing quantities (100 to 500 ng) of the Dkk1 expression plasmid or with the empty backbone vector, and the reporter activity was measured (right panel). The results were normalized for transfection efficiency and are expressed as a relative ratio of luciferase to pGL4.74 activities (denoted as relative activity). *P < 0.05 between groups. Error bars represent SDs. NS, not significant. Click here for file
