# Supplementary figures and images for: The effects of oxygen tension and antiaging factor Klotho on Wnt signaling in nucleus pulposus cells
Source: Arthritis Res Ther. 2012 May 2;14(3):R105. doi: 10.1186/ar3830 (PMC3446482; doi:10.1186/ar3830)

Supplemental Figure

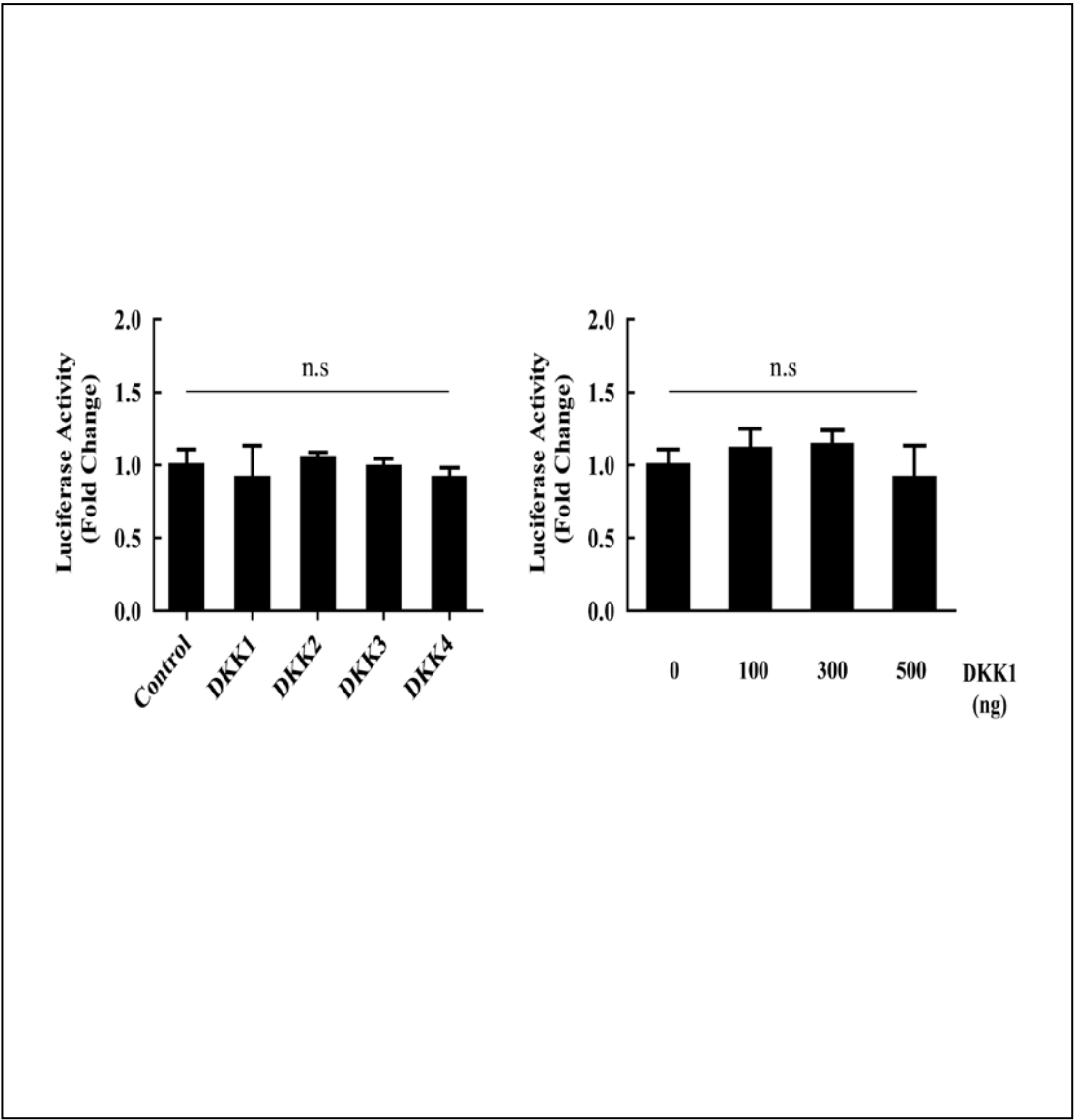

Supplement: Additional file 1 — Nucleus pulposus cells were cotransfected with the Klotho reporter plasmid along with 500 ng of Dkk1, Dkk2, Dkk3, Dkk4, or the empty backbone vector, and the reporter activity was measured (left panel). Nucleus pulposus cells were cotransfected with the Klotho promoter plasmid along with increasing quantities (100 to 500 ng) of the Dkk1 expression plasmid or with the empty backbone vector, and the reporter activity was measured (right panel). The results were normalized for transfection efficiency and are expressed as a relative ratio of luciferase to pGL4.74 activities (denoted as relative activity). *P < 0.05 between groups. Error bars represent SDs. NS, not significant. [file ar3830-S1.PDF]
